# Decreased Hering–Breuer Input-Output Entrainment in a Mouse Model of Rett Syndrome

**DOI:** 10.3389/fncir.2013.00042

**Published:** 2013-04-03

**Authors:** Rishi R. Dhingra, Yenan Zhu, Frank J. Jacono, David M. Katz, Roberto F. Galán, Thomas E. Dick

**Affiliations:** ^1^Division of Pulmonary, Critical Care and Sleep Medicine, Department of Medicine, Case Western Reserve UniversityCleveland, OH, USA; ^2^Department of Neurosciences, Case Western Reserve UniversityCleveland, OH, USA; ^3^Systems Biology and Bioinformatics Program, Case Western Reserve UniversityCleveland, OH, USA; ^4^Louis Stokes Veterans Affairs Medical Center, Case Western Reserve UniversityCleveland, OH, USA

**Keywords:** closed-loop, entrainment, vagus, Hering–Breuer reflex, *Mecp2*

## Abstract

Rett syndrome, a severe X-linked neurodevelopmental disorder caused by mutations in the gene encoding methyl-CpG-binding protein 2 (*Mecp2*), is associated with a highly irregular respiratory pattern including severe upper-airway dysfunction. Recent work suggests that hyperexcitability of the Hering–Breuer reflex (HBR) pathway contributes to respiratory dysrhythmia in *Mecp2* mutant mice. To assess how enhanced HBR input impacts respiratory entrainment by sensory afferents in closed-loop *in vivo*-like conditions, we investigated the input (vagal stimulus trains) – output (phrenic bursting) entrainment *via* the HBR in wild-type and MeCP2*-*deficient mice. Using the *in situ* perfused brainstem preparation, which maintains an intact pontomedullary axis capable of generating an *in vivo*-like respiratory rhythm in the absence of the HBR, we mimicked the HBR feedback input by stimulating the vagus nerve (at threshold current, 0.5 ms pulse duration, 75 Hz pulse frequency, 100 ms train duration) at an inter-burst frequency matching that of the intrinsic oscillation of the inspiratory motor output of each preparation. Using this approach, we observed significant input-output entrainment in wild-type mice as measured by the maximum of the cross-correlation function, the peak of the instantaneous relative phase distribution, and the mutual information of the instantaneous phases. This entrainment was associated with a reduction in inspiratory duration during feedback stimulation. In contrast, the strength of input-output entrainment was significantly weaker in *Mecp2*^−/+^ mice. However, *Mecp2*^−/+^ mice also had a reduced inspiratory duration during stimulation, indicating that reflex behavior in the HBR pathway was intact. Together, these observations suggest that the respiratory network compensates for enhanced sensitivity of HBR inputs by reducing HBR input-output entrainment.

## Introduction

Rett syndrome is caused by loss of MeCP2 function and is associated with an increase in respiratory pattern irregularity characterized by periods of forceful breathing (hyperventilation), breathing pauses, and abnormal cardiorespiratory coupling, as well as increased mean respiratory frequency (Weese-Mayer et al., 2006; Katz et al., 2012). MeCP2-deficient mice have a similar irregular breathing phenotype including increased mean respiratory frequency, increased variability in frequency, and increased frequency of apneas of both central and obstructive types (Katz et al., 2009; Voituron et al., 2010). The intrinsic neuronal mechanisms associated with these breathing alterations include widespread hyperexcitability in several respiratory areas of the brainstem including the nucleus tractus solitarius (nTS, Kline et al., 2010; Kron et al., 2012), Kölliker–Fuse nuclei (KFn, Stettner et al., 2007), locus coeruleus (Taneja et al., 2009), and ventrolateral medulla (Medrihan et al., 2008). Accordingly, therapies targeted at reducing neuronal hyperexcitability are effective in reducing the frequency of central apnea in mice (Abdala et al., 2010).

**Figure 1:**
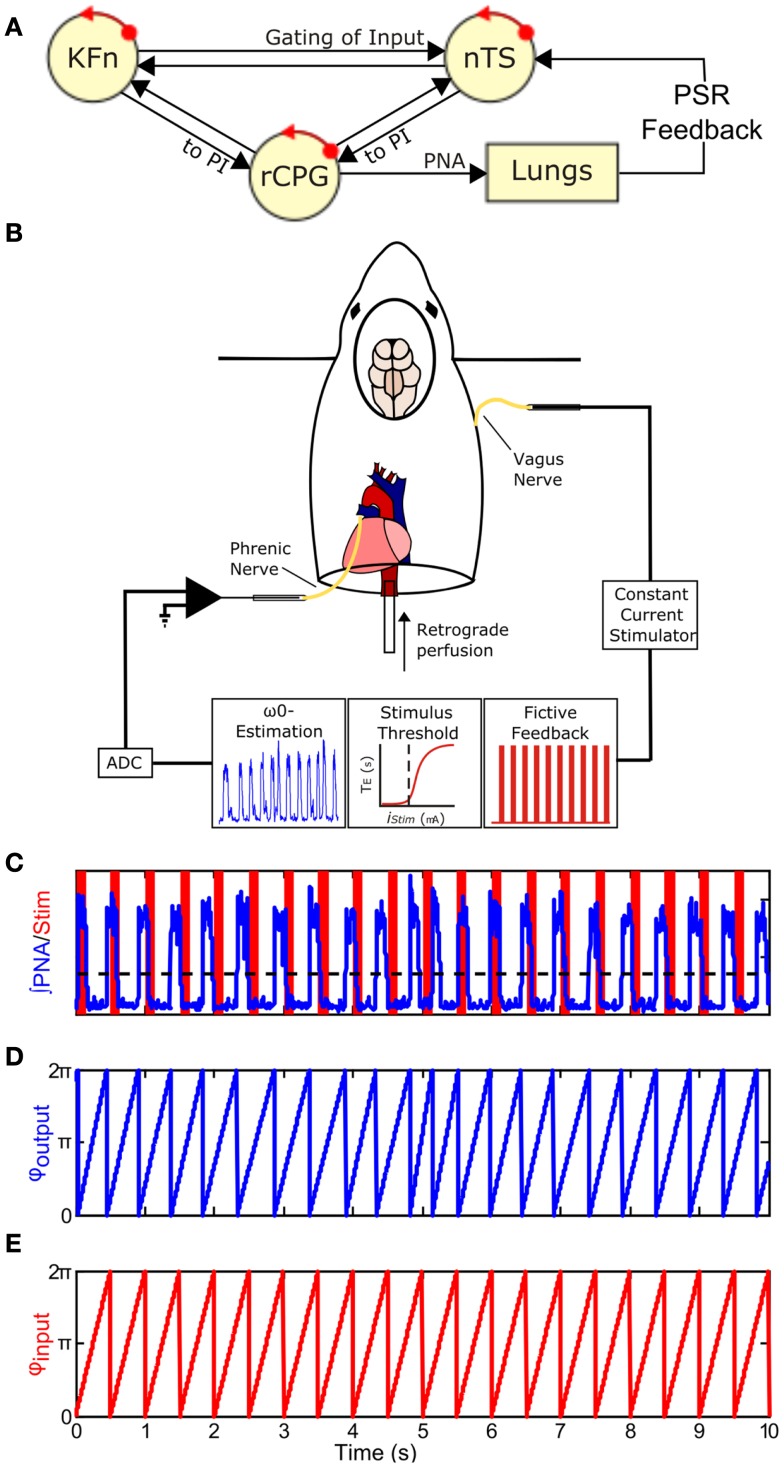
**Closing the Hering–Breuer mechanosensory feedback loop in the *in situ* arterially perfused preparation**. **(A)** Schematic of the closed-loop *in vivo* respiratory rhythm generating network. *rCPG*, respiratory CPG; *PSR*, pulmonary stretch receptor; *nTS*, nuclei of the solitary tract; *KFn*, Kölliker–Fuse nuclei. **(B)** To mimic Hering–Breuer reflex (HBR) feedback, we first estimated the intrinsic oscillation frequency, ω_0_, from an epoch of integrated phrenic nerve activity (PNA). Second, we estimated the minimum threshold to evoke the inspiratory inhibitory HBR by applying 10 s stimulus trains (20 Hz, 0.5 ms pulse-width) of increasing stimulus intensities to the contralateral vagus nerve. Once these two parameters were determined from each experimental preparation, we generated a fictive feedback input that consisted of a 2-min stimulus of rhythmic 100 ms trains (75 Hz, 0.5 ms pulse-width) whose inter-burst frequency matched ω_0_ with pulse amplitude just above the threshold for resetting. **(C)** A representative tracing of fictive feedback input (*shaded bars*) and PNA output (*trace*) from a wild-type mouse. The dashed line indicates the threshold used for *post hoc* event detection. **(D,E)** To analyze entrainment between the input and output, onset times for the two signals were extracted and used to generate the instantaneous phase time series, φ_output_(*t*) **(D)** and φ_input_(*t*) **(E)**. φ(*t*) increases linearly from 0 to 2π between events and represents the movement of each oscillator around its limit-cycle.

At the network level, MeCP2-deficiency leads to exaggerated post-inspiratory (PI) activity in vagal nerve recordings whose efferent fibers innervate the upper-airway (Stettner et al., 2007). The PI motor pattern is controlled by peripheral and pontine drives (See Figure 1A). Activation of pulmonary stretch receptor (PSR) inputs, located in the airways and lungs, send feedback encoding lung volume to the respiratory network via the vagal nerves to inhibit inspiration and facilitate expiration [the Hering–Breuer reflex (HBR); Kubin et al., 2006]. Neurons in the nTS, called pump cells, receive these vagal inputs and relay the information to medullary PI neurons as well as to the KFn causing robust inhibition of inspiration and prolongation of expiration (Berger, 1977; Ezure and Tanaka, 1996; Ezure et al., 1998, 2002). The dorsolateral pontine drive for the PI motor pattern was identified in studies in which blockade of NMDAergic transmission in the KFn after transection of the vagal nerves eliminates the inspiratory off-switch and leads to an apneustic breathing pattern (Fung et al., 1994; Ling et al., 1994). Moreover, pump cell projections to the dl pons may gate an excitatory efference copy of central pattern generator (CPG) output from late-inspiratory neurons of the pre-Bötzinger complex (Cohen and Shaw, 2004; Dick et al., 2008). Thus in the absence of vagal afferent activity, the central representation of the “pattern” is disinhibited in the pons and dl pontine activity can drive PI activity. Thereby, this circuit motif allows the network to generate a PI rhythm in the absence of closed-loop feedback control.

In MeCP2-deficient mice, because both the nTS and the KFn appear functionally hyperexcitable, it is unclear whether the excessive PI activity and the resultant respiratory pattern dysrhythmia are due to a peripheral PI mechanism involving the nTS or a pontine PI mechanism involving the KFn. To disentangle this issue, we simulate the closed-loop behavior of the network by re-introducing rhythmic vagal feedback *in situ* and measuring the ability of the wild-type versus MeCP2-deficient networks to entrain to a threshold-amplitude periodic vagal input. Entrainment to rhythmic inputs is a fundamental property of any oscillator that occurs when the weak external forcing causes the oscillator to adjust its periods to become phase-locked with the imposed rhythm. In the entrained regime, the ratio between the intrinsic oscillation frequency and the imposed forcing takes on rational values. From the phase approximation model, we know that the existence of stable coupled dynamics between the oscillator and the rhythmic forcing depends on (1) the difference in frequency between the intrinsic oscillation frequency and that of the rhythmic input and (2) the strength of the coupling. In humans and cats, respiration readily entrains to HBR inputs during mechanical ventilation (Petrillo and Glass, 1984; Graves et al., 1986). In rats, respiration can also be entrained directly by rhythmically stimulating vagal afferent nerve endings (Dutschmann et al., 2009).

In this report, we test the hypothesis that increased respiratory pattern irregularity in *Mecp2*^−/+^ mice is associated with an enhancement of respiratory entrainment by HBR inputs. While the strengthening of the coupling at the level of the nTS in MeCP2-deficient mice predicts an increase in entrainment between the CPG and the vagal input, we observed that *Mecp2*^−/+^ mice display reduced input-output entrainment consistent with a dysfunctional pontine PI mechanism that causes respiratory dysrhythmia in these mice. However, the peripheral HBR pathway is still functional because inspiratory duration decreased during rhythmic vagal stimulation.

## Materials and Methods

Experimental protocols were approved by the Case Western Reserve University Institutional Animal Care and Use Committee and were performed with strict adherence to all American Association for Accreditation of Laboratory Animal Care International (AAALAC), National Institutes of Health and National Research Council guidelines.

Experiments were performed in adult (10–12 week postnatal age), female *Mecp2*^tm1.1Jae^ mice maintained on a mixed background (129Sv, C57BL/6, Balb/c; *n* = 6 wild-type, *n* = 5 *Mecp2*^−/+^; Chen et al., 2001; Guy et al., 2001). We utilized heterozygous female mice because they more closely model the human condition in which the mutation is lethal in males and results in somatic mosaicism in females due to the stochastic nature of X-chromosome inactivation. Female *Mecp2*^tm1.1Jae^ heterozygotes show respiratory pattern irregularities like their null male littermates albeit at a later developmental time (Schmid et al., 2012). Further, a recent consortium on developing translational therapies for Rett syndrome has highlighted the importance of validating preclinical findings in heterozygous female *Mecp2* mutants (Katz et al., 2012).

To close the vagal mechanosensory feedback loop (Figure 1B), we stimulated the vagus nerve in the arterially perfused *in situ* preparation (*in situ* preparation), which is devoid of peripheral feedbacks, but maintains both an intact pontomedullary respiratory CPG and intact peripheral sensory nerve inputs (Paton, 1996). Briefly, mice were deeply anesthetized with isoflurane (1.5-3%, Piramal Healthcare, Andhra Pradesh, India). Once the mouse failed to respond to a noxious paw pinch, it was transected below the diaphragm and transferred into an ice-cold artificial cerebrospinal fluid (aCSF) bath for precollicular decerebration, cerebellectomy, and dissection of phrenic and vagal nerves. The preparation was then transferred to a recording chamber. The descending aorta was cannulated and perfused with aCSF (125 mM NaCl, 3 mM KCl, 1.25 mM KH_2_PO_4_, 2.5 mM CaCl_2_, 1.25 mM MgSO_4_, 25 mM NaHCO_3_, 10 mM d-glucose) containing 1.25% Ficoll (31°C) using a peristaltic pump (Watson and Marlow 505S, Cornwall, UK). The perfusate was continuously bubbled with a gas mixture containing 94% O_2_/6% CO_2_. Because of the small size of the descending aorta, we were not able to measure perfusion pressure accurately. Adequate perfusion of the brainstem was maintained with flows between 17 and 20 ml/min. Within minutes of cannulation, respiratory movements resumed. If the respiratory activities were initially disorganized, then a single bolus of NaCN (0.1 ml, 0.03% w/v) was delivered to stimulate the peripheral chemoreceptors and restore the eupneic-like patterning of respiratory motor output. Vasopressin was not administered during these experiments.

### Nerve recordings

Phrenic (PNA) and vagal (VNA) nerve activities were used as an index of fictive respiratory motor output. The distal end of either nerve was recorded via suction electrodes, filtered (0.003–3 kHz) and amplified (5–20 K; Grass P511, West Warwick, RI, USA), digitized (Power 1401, CED, Cambridge, UK), and stored (10 kHz sampling frequency) on a computer using Spike2 acquisition software (CED, Cambridge, UK).

### Experimental protocol

After the tuned respiratory rhythm stabilized (15–20 min), baseline activity was recorded for at least 5 min to assess differences in respiratory patterning between the genotypes and to measure the intrinsic oscillation frequency of each preparation for determining the burst frequency of fictive vagal feedback. Next, the threshold amplitude for evoking the HBR was determined by measuring the threshold for an expiratory prolongation response to a constant train of vagal stimulation (20 Hz, 10 s train duration, 0.5 ms pulse duration). The threshold amplitude was defined as the stimulus current necessary to evoke an expiratory prolongation of at least 1.5 × the baseline expiratory duration. Having determined feedback burst frequency and pulse amplitude parameters, custom scripts written in MATLAB were used to generate rhythmic event trains (75 Hz, 100 ms train duration, 0.5 ms pulse duration) whose inter-burst frequency was matched to the intrinsic PNA burst frequency (∼75–200 breaths/min). Burst stimulation was used because afferent discharge of slowly adapting PSRs are characterized by sinusoidal ramps in impulse frequency *in vivo* (Widdicombe, 1954; Luck, 1970). As these vagal PSR fibers are large and myelinated (Düring et al., 1974), the pulse duration was chosen to preferentially activate myelinated fibers.

Fictive feedback stimulation trials (2 min duration) were separated by at least 1 min of baseline activity and were repeated until the decay of the preparation caused the intrinsic oscillation frequency to drift by more than 30% from baseline which typically occurred 1.5–5 h after the resumption of PNA. During fictive feedback stimulation trials, custom Spike2 scripts transformed the MATLAB-generated event time series into a TTL output to deliver the fictive feedback to the preparation. Importantly, the fictive feedback was not triggered by the PNA. Instead, the rhythmic feedback was started irrespective of the current phase of the respiratory CPG. Nonetheless, the respiratory CPG quickly entrained the ongoing respiratory oscillation within a few cycles in wild-type mice (Figure 4A, *left panels*).

### Data analysis

Phrenic nerve activities onset and offset times were derived from a threshold crossing algorithm and visually inspected for artifacts (Figures 1C–E). Respiratory period, phase durations, variability, and apnea index were calculated from the recorded baseline epoch (*n* = 6 wild-type; 5 *Mecp2*^−/+^). Apneas were defined as respiratory cycles with duration more than 1.5 times the mean period. The Wilcoxon signed rank test was used to determine the significance of HBR-induced reduction in Ti (Figure 3).

To test our hypothesis that increased pattern irregularity in MeCP2-deficient mice is associated with alterations in the ability of the CPG to entrain to afferent feedback inputs, we characterized the input-output entrainment generated by fictive vagal feedback in *Mecp2*^−/+^ mice (*n* = 11 trials) versus wild-type littermates (*n* = 22 trials) using the cross-correlogram and several statistical measures derived from the instantaneous phase time series including the relative phase histogram, the instantaneous phase coherence, the synchronization index, and the mutual information of the instantaneous phases.

The normalized cross-correlogram, or transfer function, was computed using standard routines available in the MATLAB Signal Processing Toolbox. For computation of the cross-correlogram, the input signal was represented by a square-wave function with a pulse-width equal to the train duration. Before computing the cross-correlogram, both input and output signals were scaled between 0 and 1 and DC-removed. The maximum of the cross-correlogram was used as an index of the strength of input-output coupling.

The instantaneous phase time series was determined from onset times of the phrenic or input signals, *t_k_*. The instantaneous phase, φ(*t*), which is assumed to grow linearly in time within each cycle, was defined according to the following equation:

(1) $$\begin{array}{l} \varphi \left(t\right)=2\pi \frac{t-t_{k}}{t_{k}-t_{k+1}}+2\pi ,\;\;\;t_{k}<t<t_{k+1} \end{array}$$

where *t_k_* is the time of the *k*-th event, and *t*_k+1_ is the time of the next event.

From the instantaneous phase time series’, the instantaneous relative phase difference time series, φ_output_ − φ_input_, was computed. Histograms of the instantaneous relative phase were computed using 21 bins over the range 0–2π and scaled by the number of samples to determine the probability of a given instantaneous relative phase. The deviation from a uniform distribution was determined using the Rayleigh test for circular uniformity. The maximum of the instantaneous relative phase histogram was used as a measure of input-output coupling.

To define the regions in the instantaneous relative phase time series that were associated with strong input-output entrainment, we computed the phase coherence of the relative phases. The phase coherence is a windowed statistic that measures the squared magnitude of the mean phase angle:

(2) $$\gamma \left(t\right)={∥\frac{1}{N}\overset{t}{\underset{i=t-w}{\sum }}e^{i\left[\varphi_{\text{output}}-\varphi_{\text{input}}\right]}∥}^{2}$$

The phase coherence was computed with a 3s window. The phase coherence yields a value between 0 and 1. Values near zero are not phase-locked, whereas values closer to 1 indicate the presence of phase-locking. To measure the latency to entrainment, the duration of entrainment and number of phase slips, we used a phase coherence threshold of 0.9.

The synchronization index, or phase-locking value, also maps the circular distribution of relative instantaneous phase onto the unit circle. The magnitude of the synchronization index is proportional to the degree of input-output entrainment. The synchronization index is computed via the following equation:

(3) $$\begin{array}{l} \gamma_{\left(n,\;m\right)}=\left|\left\langle e^{i\left[n\varphi_{\text{output}}-m\varphi_{\text{input}}\right]}\right\rangle \right| \end{array}$$

for any *n:m* coupling. In the present study, the input oscillation frequency was chosen such that only 1:1 coupling was observed.

Mutual information is a measure of statistical dependence in a pair of time series. We used mutual information of the instantaneous phases in combination with surrogate data testing to quantify input-output entrainment and to allow for a statistical determination of the significance of the observed phase-locking. The mutual information index is defined according to the following equation:

(4) $$I\left(\varphi_{\text{input}},\;\varphi_{\text{output}}\right)=H\left(\varphi_{\text{input}}\right)+H\left(\varphi_{\text{output}}\right)-H\left(\varphi_{\text{input}}|\varphi_{\text{output}}\right)$$

where *H*(φ*_X_*) is the entropy of time series φ*_X_* computed from the individual probability histogram, and *H*(φ*_X_*|φ*_Y_*) is the conditional entropy of time series φ*_X_* and φ*_Y_* computed from the joint probability histogram. The entropy of either distribution is computed according to:

(5) $$H\left(\varphi_{X}\right)=-\overset{L}{\underset{k=1}{\sum }}P\left(\varphi_{X}\left(k\right)\right)\ln P\left(\varphi_{X}\left(k\right)\right)$$

where *L* is the number of bins in the histogram and *P*(φ*_X_*(*k*)) is the probability of observing φ*_X_* in bin *k*. Note that because the mutual information index is sensitive to the number of bins *L*, we consistently used 50 bins in the generation of all histograms to allow for comparisons across experiments.

To determine the significance of the observed input-output coupling, a surrogate data testing scheme was needed to represent the null hypothesis of independent pairs of oscillatory activity. To generate bootstrapped distributions of the null hypothesis, we randomized the inter-event intervals of both the input and output before computing the instantaneous phases (500 surrogates/trial). The mutual information of these surrogate time series’ was then computed to generate the bootstrapped mutual information histogram. The observed mutual information value of the coupling was considered significant if it fell above the 99% confidence interval of the bootstrap distribution. This criteria served as the basis for the identification of intermediate and severe *Mecp2*^−/+^ entrainment defects discussed in Figures 4 and 5.

All data were expressed as mean ± SEM. Unless stated otherwise, we applied one-way repeated measures ANOVA to determine the significance of the results. If significant, we used a Bonferroni *post hoc* test to determine specific differences.

## Results

**Figure 2:**
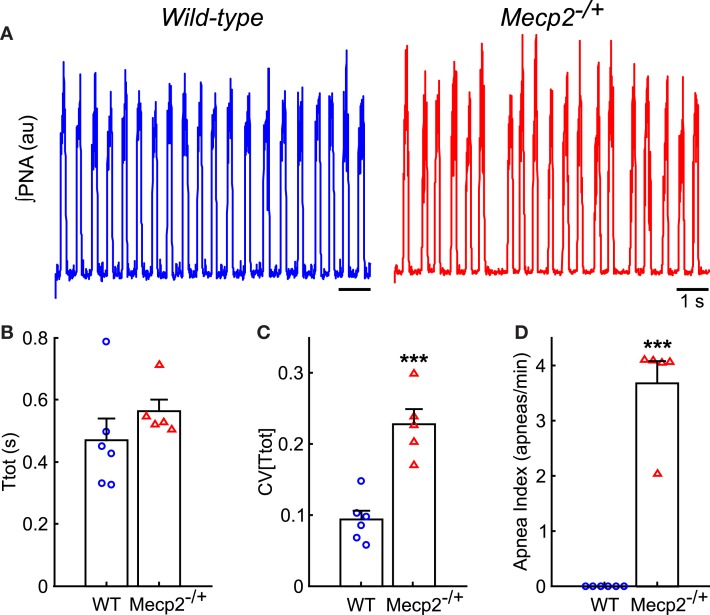
***Mecp2*^−**/+**^ mice have increased respiratory pattern variability *in situ***. **(A)** Representative traces of ∫PNA during baseline recordings show the presence of spontaneous apneas in *Mecp2*^−/+^, but not wild-type mice. **(B)** Respiratory period does not differ between wild-type and *Mecp2*^−/+^ mice *in situ*. **(C)** *Mecp2*^−/+^ mice have increased variability in the respiratory period. **(D)** *Mecp2*^−/+^ mice have an increased frequency of spontaneous apnea. ***p < 0.001.

Representative traces of PNA from wild-type and *Mecp2*^−/+^ baseline breathing patterns are shown in Figure 2A. The duration of the respiratory period was not significantly different between genotypes (Figure 2B), but the variability of the respiratory period [CV(Ttot)] was greater in *Mecp2*^−/+^ than wild-type mice (*Mecp2*^−/+^, 0.23 ± 0.02 versus wild-type, 0.09 ± 0.01; *p* < 0.001; Figure 2C). The irregularity of the pattern was characterized by a higher frequency of spontaneous apnea in *Mecp2*^−/+^ versus wild-type mice (*Mecp2*^−/+^, 3.7 ± 0.4 apneas/min versus wild-type, 0 ± 0 apneas/min; *p* < 0.001; Figure 2D). The intrinsic respiratory oscillation in *Mecp2*^−/+^ mice had strong vagal efferent activity (*data not shown*) consistent with patterns reported previously (Stettner et al., 2007; Abdala et al., 2010).

**Figure 3:**
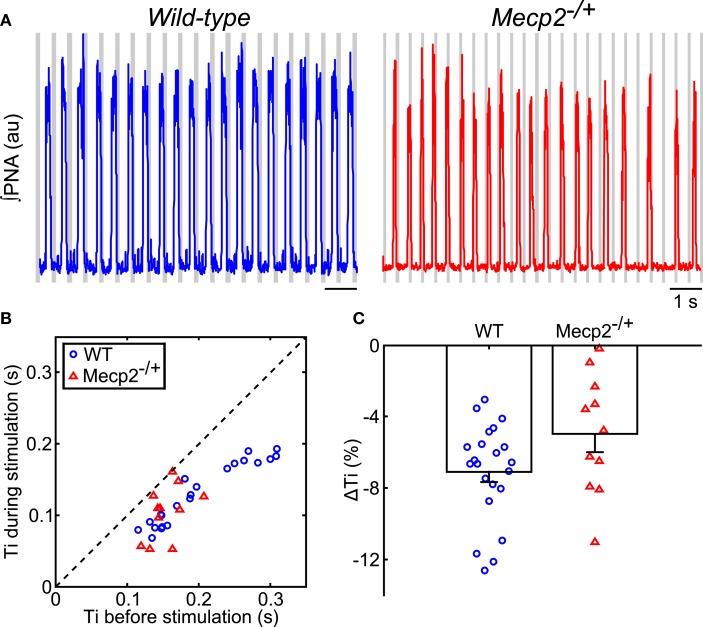
**Hering–Breuer feedback stimulation decreases inspiratory phase duration of the respiratory pattern in wild-type and *Mecp2*^−**/+**^ mice**. **(A)** Representative traces of ∫PNA (traces) during rhythmic vagal stimulation. Gray bars indicate the fictive feedback stimulation. **(B)** Rhythmic stimulation significantly decreases inspiratory duration in both wild-type and *Mecp2*^−/+^ mice (wild-type, *p* = 4.8 × 10^−7^; *Mecp2*^−/+^, *p* = 9.8 × 10^−4^). **(C)** HBR-dependent reduction in inspiratory duration tends to be smaller in *Mecp2*^−/+^ versus wild-type mice (*p* = 0.065).

During rhythmic vagal nerve stimulation (Figure 3A), inspiratory duration (Ti) decreased in both wild-type and *Mecp2*^−/+^ mice as indicated by the pair-wise deviation from the line of identity (wild-type: baseline, 0.20 ± 0.01 s versus stimulation, 0.13 ± 0.001 s, *p* = 5 × 10^−7^; *Mecp2*^−/+^: baseline, 0.15 ± 0.01 s versus stimulation, 0.10 ± 0.01 s, *p* = 5 × 10^−4^; Figure 3B) consistent with the role of this peripheral sensory modality in the inspiratory off-switching mechanism. Further, the reduction in Ti induced by vagal stimulation tended to be smaller in *Mecp2*^−/+^ versus wild-type mice, though this difference was not significant (*Mecp2*^−/+^, −5.0 ± 1.0% versus wild-type, −7.1 ± 0.1%, *p* = 0.065; Figure 3C).

**Figure 4:**
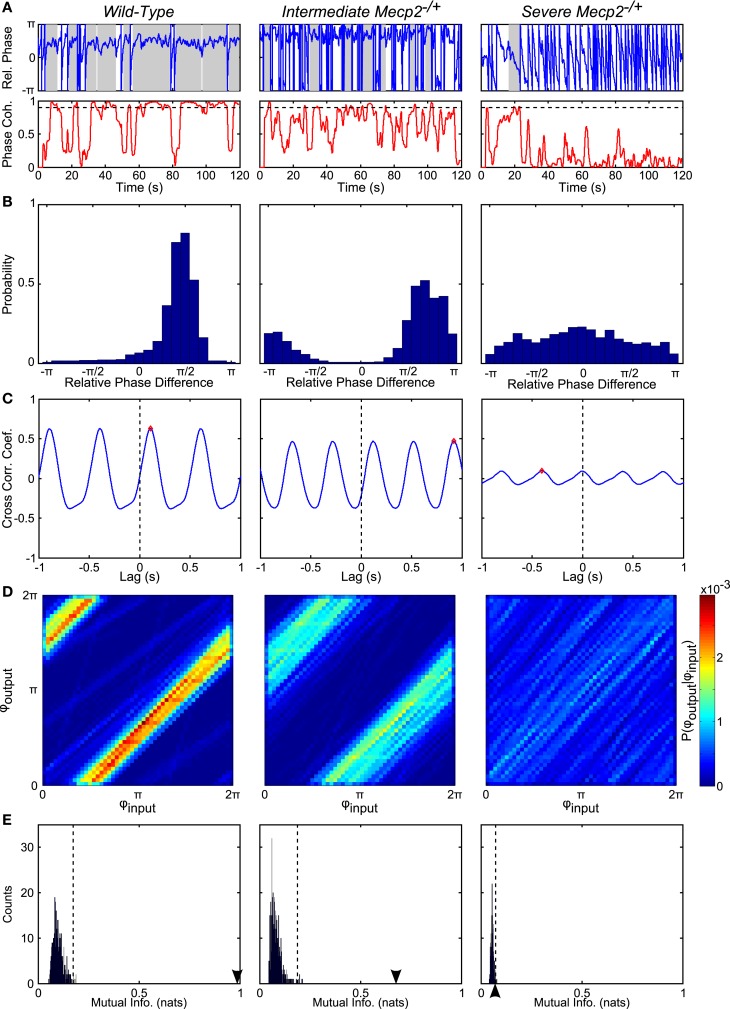
**Representative analyses show reduced phase-locking in *Mecp2*^−/+^ compared to wild-type mice**. **(A)** From the instantaneous phase time series, φ_output_ and φ_input_, we computed the relative instantaneous phase difference time series, φ_output_ − φ_input_ (*top panels*). Regions with near zero slope are indicative of input-output entrainment, which was more frequently observed in wild-type relative to *Mecp2*^−/+^ mice. Phase coherence (*bottom panels*) is a windowed measure that captures the strength of input-output entrainment. A threshold of 0.9 (*dashed line in bottom panels*) was used to determine windows with significant input-output phase-locking (*shaded regions in top panels*). *Mecp2*^−/+^ mice had increased latency to and reduced duration of entrainment relative to wild-type mice. **(B)** Representative histograms of the instantaneous phase difference are shown for a wild-type (left panel) and *Mecp2*^−/+^ mice (*middle and right panels*). In all cases, even the severe *Mecp2*^−/+^ mice, the distribution was significantly different from a uniform circular distribution as determined by the Rayleigh test for circular non-uniformity. **(C)** Representative cross-correlograms are shown for a wild-type (left panel), intermediate *Mecp2*^−/+^ (middle panel), and severe *Mecp2*^−/+^ (right panel) mice. The maximum of the cross-correlogram was reduced in *Mecp2*^−/+^ versus wild-type mice. **(D)** Representative joint probability histograms of the instantaneous input- and output-phases depict the strength of input-output entrainment in the intensity of banding. Entrainment was strong in wild-type and intermediate *Mecp2*^−/+^ preparations, but was abolished in severe *Mecp2*^−/+^ mice. **(E)** The significance of the observed input-output entrainment was determined by generating bootstrap distributions of the mutual information of the instantaneous phases. Surrogate instantaneous phase time series were generated by shuffling the input- and output-inter-event intervals before determining the phase. The observed value of the mutual information is indicated by the arrowheads on the abscissa. The upper-bound of the 99% confidence interval is indicated by dashed vertical lines. Wild-type and intermediate *Mecp2*^−/+^ mice always showed significant input-output entrainment, whereas severe *Mecp2*^−/+^ mice did not have significant input-output entrainment.

**Figure 5:**
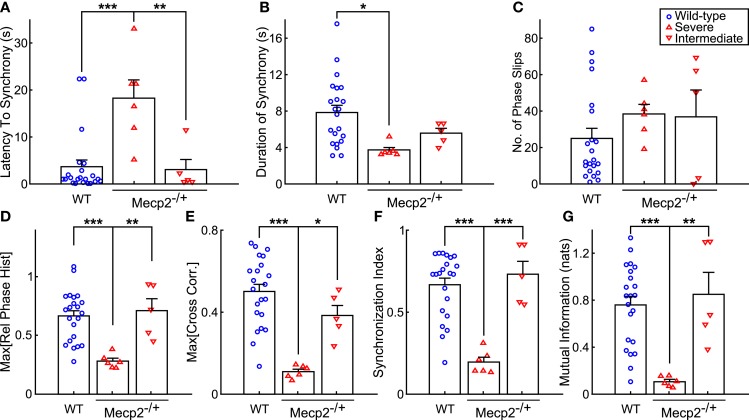
***Mecp2*^−**/+**^ mice show reductions in input-output phase-locking across multiple entrainment measures**. **(A)** Severe *Mecp2*^−/+^ mice had significantly increased latency to input-output entrainment from the start of rhythmic stimulation compared to both wild-type and intermediate *Mecp2*^−/+^ mice. **(B)** Severe *Mecp2*^−/+^ mice had significantly shorter bouts of input-output phase-locking compared to wild-type mice. **(C)** Severe and intermediate *Mecp2*^−/+^ mice had a mild tendency for increased phase slips. **(D)** Relative to wild-type and intermediate mice, the maximum of the phase histogram was significantly less in severe *Mecp2*^−/+^ mice. **(E)** The maximum of the cross-correlation function was significantly reduced in severe *Mecp2*^−/+^ compared to wild-type and intermediate *Mecp2*^−/+^ mice. **(F)** For the group, severe *Mecp2*^−/+^ mice had a significantly lower synchronization index relative to both wild-type and intermediate *Mecp2*^−/+^ mice. **(G)** Analysis of the mutual information of the instantaneous phases also revealed significantly weaker input-output entrainment in severe *Mecp2*^−/+^ compared to wild-type and intermediate *Mecp2*^−/+^ mice. *p < 0.05, **p < 0.01, ***p < 0.001.

To test our hypothesis, we analyzed the input-output phase-locking between rhythmic vagal stimulation and the central respiratory oscillation (Figures 4 and 5). To characterize the significance of input-output coupling in individual trials, we relied on the mutual information index applied in conjunction with a bootstrapping approach wherein surrogate time series were generated via shuffling the inter-burst intervals (Figures 4D,E and 5A–G; See Materials and Methods). As expected, wild-type mice had significant input-output entrainment in all trials (19/19 trials). By contrast, entrainment varied in *Mecp*2^−/+^ mice: a severe group (6/11 trials) had a complete loss of input-output coupling; and an intermediate group (5/11 trials) had weak, but still significant input-output phase-locking (Figure 4). To fully characterize the changes in input-output coupling, we analyzed the relative phase difference time series (Figures 4A,B), the input-output cross-correlogram (Figure 4C), and the mutual information of the instantaneous phases (Figures 4D,E).

Representative traces of the relative phase time series during closed-loop stimulation are presented in Figure 4A (*top panels*). Entrainment between input and output is observed as epochs with a slope near 0, whereas sharp spikes in the time series are indicative of phase slips (Figure 4A). To characterize the duration and latency to input-output phase-locking, we measured the phase coherence, or mean phase angle on the unit circle, using a sliding windowed algorithm (Figure 4A, *bottom panels*). Phase-locked epochs within the trial were indicated by contiguous time regions where the phase coherence was >0.9 (Figure 4A, *shaded regions in top panels, horizontal lines in bottom panels*). *Mecp2*^−/+^ mice had a strong tendency toward greater latency to input-output phase-locking from the beginning of a stimulation trial relative to wild-type mice (*Mecp2*^−/+^, 11.3 ± 3.3 s versus wild-type, 3.7 ± 1.4 s, *p* = 0.07). Further, severe *Mecp2*^−/+^ mice had a significantly greater latency to phase-locking compared to intermediate or wild-type mice (severe, 18.2 ± 3.9 s versus wild-type, 3.7 ± 1.4 s, *p* < 0.001, versus intermediate, 3.0 ± 2.1 s, *p* < 0.01, Figure 5A). Wild-type mice had longer durations of stable entrainment relative to *Mecp2*^−/+^ mice (wild-type, 7.8 ± 0.8 s versus *Mecp2*^−/+^, 4.5 ± 0.4 s, *p* < 0.01, versus severe, 3.7 ± 0.3 s, *p* < 0.05, Figure 5B). Compared to the severe group, intermediate *Mecp2*^−/+^ mice had a tendency for longer durations of entrainment, but this was not significant. Further, *Mecp2*^−/+^ mice also showed a mild tendency for an increased number of phase slips during stimulation trials relative to wild-type mice (*Mecp2*^−/+^, 37.5 ± 6.9 slips/trial versus wild-type, 24.9 ± 5.4 slips/trial, *p* = 0.37, Figure 4C). Finally, the severe *Mecp2*^−/+^ group had fewer bouts of input-output phase-locking (3/6 trials), whereas the intermediate *Mecp2*^−/+^ group consistently showed short bouts of input-output phase-locking (5/5 trials).

Representative relative phase histograms are shown in Figure 4B. A preferred relative phase between the vagal input and phrenic output was observed in both wild-type and the intermediate *Mecp2*^−/+^ group, but not the severe *Mecp2*^−/+^ group, which had a more uniform circular distribution of relative phases during rhythmic stimulation trials. However, all distributions had a measurable directionality as indicated by the Rayleigh test for deviance from circular uniformity indicative of a functional HBR. For the group, the maximum of the relative phase histogram and the synchronization index – the mean resultant vector of the relative phase time series – were both greater in wild-type relative to *Mecp2*^−/+^ mice [Max (Rel. Phase Hist.): wild-type, 0.66 ± 0.05 versus *Mecp2*^−/+^, 0.48 ± 0.08, *p* < 0.05, wild-type versus severe, 0.28 ± 0.02, *p* < 0.001, severe versus intermediate, 0.71 ± 0.10, *p* < 0.01, Figure 5D; and synchronization index: wild-type, 0.67 ± 0.04 versus *Mecp2*^−/+^, 0.44 ± 0.09, *p* < 0.05, wild-type versus severe, 0.19 ± 0.03, *p* < 0.001, severe versus intermediate, 0.72 ± 0.08, *p* < 0.001, Figure 5F].

We also computed the transfer function of the system during rhythmic feedback stimulation as a measure of input-output phase-locking (Figure 4C). Cross-correlograms were periodic with the successive peaks and troughs decaying monotonically with increasing lag. While the qualitative structure of the functions was not changed between wild-type and *Mecp2*^−/+^ mice, the peak of the transfer function decreased in *Mecp2*^−/+^ mice [Max (Cross-correlation): *Mecp2*^−/+^, 0.23 ± 0.05 versus wild-type, 0.50 ± 0.04, *p* < 0.001]. Wild-type and intermediate mice also significantly differed from the severe group [Max (Cross-correlation): wild-type, 0.23 ± 0.05 versus severe, 0.11 ± 0.01, *p* < 0.001, severe versus intermediate, 0.38 ± 0.05, *p* < 0.05, Figure 5E].

Finally, we characterized the joint probability distribution functions of the instantaneous input- and output-phases by computing their mutual information, which quantifies the general dependence between the phase of the input and the phase of the output. To determine the significance of the observed entrainment, we performed bootstrap analyses by shuffling the inter-event intervals and re-computing the mutual information of the instantaneous phases. Phase-locking, characterized by clear banding in the joint probability distribution function, was observed in the wild-type and intermediate *Mecp2*^−/+^ group (Figure 4D, left and center panels respectively), whereas the uniform joint probability distribution function of the severe *Mecp2*^−/+^ group (Figure 4D, right panel) reflected the drifting of the instantaneous phases. Representative bootstrapped mutual information histograms were roughly Gaussian, though bounded >0 because of the rarity of obtaining a perfectly uniform joint probability distribution (Figure 4E). The input-output entrainment was considered significant if the observed mutual information was greater than the 99% confidence interval of the bootstrap distribution. Five of 11 *Mecp2*^−/+^ had significant entrainment according to the bootstrap results and were thereby classified as the intermediate *Mecp2*^−/+^ phenotype. For the group, the mutual information of the instantaneous phases was greater in wild-type relative to *Mecp2*^−/+^ mice (mutual information: wild-type, 0.76 ± 0.07 versus *Mecp2*^−/+^, 0.44 ± 0.14, *p* < 0.05). Severe mice also had significantly weaker entrainment as measured by the mutual information of the instantaneous phases compared to both wild-type and intermediate mice (severe, 0.11 ± 0.02 versus wild-type, 0.76 ± 0.07, *p* < 0.001, versus intermediate, 0.85 ± 0.19, *p* < 0.01, Figure 5G).

## Discussion

Imposing rhythmic vagal feedback stimulation in the *in situ* preparation decreased Ti and evoked robust bouts of input-output phase-locking in wild-type mice. Contrary to our hypothesis, *Mecp2*^−/+^ mice had significantly weaker input-output phase-locking though the decrease in Ti during vagal feedback stimulation suggested that the HBR was still intact. Using mutual information with bootstrapped surrogate distributions to evaluate significant input-output entrainment, *Mecp2*^−/+^ mice were separated into intermediate and severe entrainment phenotypes consistent with the mosaic expression of MeCP2. Severe *Mecp2*^−/+^ mice completely lost input-output entrainment, where as intermediate *Mecp2*^−/+^ mice had significant input-output entrainment, but was weaker relative to wild-type mice. Together, our findings identify a compensatory adaptation of the MeCP2-deficient respiratory network that decouples the respiratory rhythm from vagal feedback inputs.

### Technical considerations

In the present study, we assessed the ability of the isolated adult brainstem respiratory network to entrain to rhythmic vagal stimulation as a model of the *in vivo* closed-loop condition. As noted in the introduction, the presence of stable entrainment depends on two factors: (1) the frequency difference between the oscillators, e.g., the weakly coupled oscillator network that comprises the respiratory rhythm generator, and the periodic input, e.g., the rhythmic vagal stimulation; and (2) the strength of the coupling between the oscillator and the input. We controlled for frequency differences by tuning the fictive vagal feedback frequency to that of each preparation. Thus, even though the *Mecp2*^−/+^ mice used in this study had a slightly increased period *in situ*, this did not prevent stable entrainment because they received a suitably slower fictive feedback input. Similarly, we controlled for differences in the strength of coupling stimulating the vagus nerve at the threshold for evoking HBR-like responses. Additionally, for the purpose of investigating closed-loop control of respiratory behavior, the utilization of the*in situ* preparation was critical because it maintains an intact pontomedullary axis, spares sensory afferent, and motoneuronal efferent pathways, and produces an *in vivo*-like respiratory rhythm allowing re-introduction of fictive vagal feedback without confounding changes in chemosensory and baroreceptor afferent pathways (Paton, 1996). Further, the absence of anesthesia was particularly important for investigating the MeCP2-deficient breathing phenotype as breathing arrhythmias in these mice are reduced by anesthetics (Viemari et al., 2005; Abdala et al., 2010).

A key caveat of our study is that fictive feedback was delivered by stimulating whole vagal nerve bundles which contain fibers from three types of pulmonary receptors: slowly adapting receptor (SAR) fibers, rapidly adapting receptor fibers, and C-fibers. In rats, SAR and RAR fibers can be stimulated preferentially because they are thick and myelinated. Accordingly, a short pulse duration and a low stimulus current were chosen to activate myelinated rather than unmyelinated fibers. Moreover, vagal stimulation, unlike lung inflation, has been shown to activate PI output recorded from the pharyngeal branch of the vagus (Hayashi and McCrimmon, 1996). However, in this study, the authors observed similar effects on inspiratory and expiratory phase durations when comparing vagal stimulation- and lung inflation-induced HBRs suggesting that functional effects of vagal stimulation and lung inflation on network output are similar enough to warrant such comparison. Moreover, these authors went on to use this same paradigm of vagal stimulation to identify neurons in the ventrolateral medulla whose activities are modulated in a paucisynaptic fashion to mediate the HBR (Hayashi et al., 1996). Similarly, in the present study and others, we observe HBR-like responses to vagal stimulation consistent with those reported for lung inflation (Karczewski et al., 1980; Budzinska et al., 1981; Siniaia et al., 2000; Dutschmann et al., 2009). Moreover, the respiratory network readily entrains to rhythmic vagal stimulation in rats (Dutschmann et al., 2009) as well as our fictive feedback trials in wild-type mice. However, from the present findings, we can conclude only that the vagal feedback entrainment behavior is lost in the MeCP2-deficient respiratory network without further experiments to dissect the mechanistic basis for the loss of functional connectivity between input and output.

### Relevance to respiratory abnormalities in rett syndrome

Given previous findings of hyperexcitablity in TS-nTS synapses and exaggerated HBR-like responses to vagal stimulation, we hypothesized that entrainment between peripheral feedbacks and the respiratory rhythm should be enhanced in MeCP2-deficient mice (Stettner et al., 2007; Kline et al., 2010; Song et al., 2011). Instead, by using rhythmic vagal stimulation, we observed a reduction in input-output entrainment suggesting that despite the exaggerated HBR responses, observed during vagal stimulation with continuous trains, vagal feedback appears to be filtered by a compensatory adaptation of the network such that rhythmic inputs above the threshold for eliciting phase resetting have little consistent effect on the respiratory rhythm. Previous findings support a role for dysfunctional postnatal maturation of the KFn in the loss of phase-locking with afferent feedbacks. As mentioned earlier, the KFn is a key determinant of the PI motor pattern because local blockade of NMDAergic transmission results in apneusis (Fung et al., 1994; Ling et al., 1994; Dutschmann and Herbert, 2006). Moreover, the KFn has reciprocal connectivity with the vl NTS such that the KFn can gate the influence of peripheral inputs on the respiratory rhythm (Herbert et al., 1990; Ezure et al., 2002; Dutschmann and Dick, 2012). Dutschmann et al. (2009) demonstrated that after postnatal maturation, repeated trials of vagal stimulation leads to an anticipatory transition to from inspiration to expiration that precedes the arrival of vagal stimuli. This learning process also depended on NMDAergic transmission, which has been shown to mature postnatally with a similar developmental time course (Kron et al., 2008). In MeCP2-deficient mice, glutamate microinjection in the KFn results in an exaggerated PI apnea (Stettner et al., 2007). Moreover, the response to constant vagal stimulation shows a loss of habituation and desensitization (Stettner et al., 2007; Song et al., 2011), which have previously been shown to depend on the KFn (Siniaia et al., 2000). These data lead to the speculation that ponto-vagal interactions may be the critical factor mediating the irregular breathing pattern in *Mecp2*^−/+^ mice.

Alternatively, the noise in the respiratory network is likely a critical factor in preventing stable coupling between the network and its peripheral feedbacks in MeCP2-deficient mice. From the phase approximation model, we know that noise reduces the parameter space associated with stable phase-locked dynamics between an oscillator and a rhythmic forcing. Thus, if the respiratory rhythm generator itself is more variable, this would prevent stable entrainment with the dynamics of the periphery. Accordingly, variability in the respiratory rhythm recorded from MeCP2-deficient mice is present in the absence of peripheral feedbacks: both in the isolated CPG *in vitro* (Viemari et al., 2005), as well as *in situ* where the network is intact, but functionally isolated from peripheral feedback (Stettner et al., 2007; Abdala et al., 2010).

### Phase synchronization measures for investigating closed-loop respiratory behavior

Over the past decade, several reports have explored the restoration of rhythmic vagal feedback in reduced preparations. Mellen and Feldman (2000) first re-introduced phasic lung inflation in the *en bloc* preparation which was modified to maintain the lungs and vagal nerve pathways. Though they established that the medullary components were sufficient to evoke the HBR mechanism as evidenced by a reduction in inspiratory duration, their preparation did not include pontine components that also modulate the PI motor pattern. Utilization of the *in situ* preparation overcomes this limitation and confirmed that phasic lung inflation reduces inspiratory duration and increases respiratory frequency (Harris and St-John, 2005). Importantly, these earlier approaches utilized closed-loop stimulation wherein lung inflation was triggered by the onset of PNA. Interactions between the respiratory rhythm and rhythmic HBR feedback inputs are also apparent when the stimulation is not coupled with the motor output as in the present study. As mentioned above, Dutschmann et al. (2009) used this latter approach to demonstrate that the pontine control of PI activity is subject to postnatal maturation and depends on NMDAergic transmission in the KFn. However, in this report, only cycle-triggered averaging was used to demonstrate the presence of functional input-output entrainment. Our approach extends this and earlier methodologies by introducing robust measures of phase synchronization that are necessary to evaluate entrainment. In the present study, the use of surrogate data sets in concert with mutual information of the instantaneous phases as a test statistic permitted assessment of significant phase-locking between the rhythmic input and respiratory motor output in individual stimulation trials. Additionally, the use of instantaneous relative phase and phase coherence allowed us to identify bouts of entrainment within individual stimulation trials. Applying our improved methodology to MeCP2-deficient mice revealed intermediate and severe decrements in input-output entrainment that were not recognized previously. In the future, our approach could be extended with a data-driven modeling approach based on the generic phase oscillator model by incorporating multiple central field recordings within the pontomedullary respiratory column to understand changes in the directionality of coupling within the respiratory network during HBR feedback stimulation (Zhu et al., 2013).

On a more general level, the findings of this study raise the possibility that the respiratory rhythm is tuned to the dynamics of the periphery. In locomotor CPGs, central oscillations are coupled to the dynamics of the limb at the a frequency between the intrinsic frequencies of the neural oscillator and of the physical limb system (Hatsopoulos, 1996; Chiel and Beer, 1997). A hallmark of this phenomenon, resonance tuning of a CPG, is that the frequency of the instrinsic oscillation is reduced in the absence of feedback (Pearson et al., 1983). Mathematically, considering the most general model of phase-coupled oscillators, this property is a consequence of the fact that the coupling between oscillators is both weak and additive with respect to the intrinsic oscillation frequency. While researchers have not previously considered resonance tuning in the context of respiration, the intrinsic respiratory oscillation frequency is reduced after removal of PSR (Stella, 1938; Dhingra et al., 2011) or peripheral chemoreceptor (Eldridge, 1974; Miller and Tenney, 1975; Hayashi et al., 1983) afferent inputs in cats and rodents. Further investigation of whether resonant tuning of respiratory dynamics may be critical for rhythmogenesis is warranted because the presence of excitatory feedback changes membrane current dynamics underlying phase switching in models of feedback-coupled locomotor CPGs (Spardy et al., 2011). Even though CPGs are defined by their ability to transform a constant drive into a rhythmic output without sensory feedback, in the intact animal, output rhythms are modulated continuously in a closed-loop fashion by peripheral afferent oscillations that, contrary to the assertions of the last decades, may be central to the rhythm generating mechanism.

## Conflict of Interest Statement

The authors declare that the research was conducted in the absence of any commercial or financial relationships that could be construed as a potential conflict of interest.
